# 2010 ACVIM Small Animal Consensus Statement on Leptospirosis: Diagnosis, Epidemiology, Treatment, and Prevention

**DOI:** 10.1111/j.1939-1676.2010.0654.x

**Published:** 2011-01

**Authors:** JE Sykes, K Hartmann, KF Lunn, GE Moore, RA Stoddard, RE Goldstein

**Affiliations:** Department of Medicine & Epidemiology, University of CaliforniaDavis, Davis, CA; Department of Small Animal Medicine, Ludwig Maximilian UniversityMunich, Germany; Department of Clinical Sciences, Colorado State UniversityFort Collins, CO; Department of Comparative Pathobiology, Purdue UniversityWest Lafayette, IN; Bacterial Special Pathogens Branch, Centers for Disease Control and PreventionAtlanta, GA; Department of Clinical Sciences, Cornell University College of Veterinary MedicineIthaca, NY

**Keywords:** Hepatitis, *Leptospira interrogans*, Nephritis, Zoonosis

## Abstract

This report offers a consensus opinion on the diagnosis, epidemiology, treatment, and prevention of leptospirosis in dogs, an important zoonosis. Clinical signs of leptospirosis in dogs relate to development of renal disease, hepatic disease, uveitis, and pulmonary hemorrhage. Disease may follow periods of high rainfall, and can occur in dogs roaming in proximity to water sources, farm animals, or wildlife, or dogs residing in suburban environments. Diagnosis is based on acute and convalescent phase antibody titers by the microscopic agglutination test (MAT), with or without use of polymerase chain reaction assays. There is considerable interlaboratory variation in MAT results, and the MAT does not accurately predict the infecting serogroup. The recommended treatment for optimal clearance of the organism from renal tubules is doxycycline, 5 mg/kg PO q12h, for 14 days. Annual vaccination can prevent leptospirosis caused by serovars included in the vaccine and is recommended for dogs at risk of infection.

Leptospirosis is a zoonotic bacterial disease with a worldwide distribution, and is an emerging infectious disease in humans^1^ and in dogs.^2^,^3^ It has been reported in over 150 mammalian species.^4^*Leptospira* spp. are thin, motile spirochetes with a hook-shaped end. Both saprophytic and pathogenic species exist in nature. Saprophytic species, such as *Leptospira biflexa*, live in water and soil and do not infect animals. Leptospires phylogenetically and pathogenically intermediate to these 2 groups also have been identified in humans and animals, but not yet in dogs.^5^,^6^ There are over 250 pathogenic serovars based on differences in the carbohydrate component of the bacterial lipopolysaccharide.^1^,^4^ Different serovars are adapted to different wild or domestic animal reservoir hosts, and thus serovar recognition has epidemiologic importance. Serovars are further grouped into antigenically related serogroups (Table 1). Immunity to leptospires is serogroup specific, and knowledge of serogroups that commonly cause disease within a particular geographic region is important for vaccine development.

**Table 1:** Leptospiral serogroups and serovars isolated from dogs suspected to have leptospirosis, or that induce disease after experimental inoculation of dogs

Species	Serogroup	Serovar	Country	References
*Leptospira interrogans*	Icterohaemorrhagiae	Icterohaemorrhagiae	USA, France	21,70–72,74–79
	Canicola	Canicola	India, USA	21,65,66,74–79
	Pomona	Pomona	USA	17
	Australis	Bratislava	USA	81
	Sejroe	ND	Germany	82
	Autumnalis	Autumnalis	India, France	62,80
	Djasiman	Buenos Aires	Argentina	29
	Ballum	Ballum	USA	49
*Leptospira kirschneri*	Grippotyphosa	Grippotyphosa	USA	18,48
*Leptospira noguchii*	Australis	ND	Brazil	8

Disease in dogs is caused primarily by *Leptospira interrogans* and *Leptospira kirschneri*. *Leptospira wolfii* was identified in dogs in Iran, but its role as a canine pathogen requires further study.^7^*Leptospira noguchii* was isolated from a sick dog in Brazil.^8^ The most common serovars thought to infect dogs before the introduction of leptospirosis vaccines 30 years ago were Icterohaemorrhagiae and Canicola. Since the introduction of bivalent Icterohaemorrhagiae and Canicola vaccines, more widespread involvement of additional serovars has been suspected, including Grippotyphosa, Pomona, Bratislava, and Autumnalis.^9^–^14^ Increased recognition of leptospirosis associated with these serovars may have resulted partly from increased testing, because a broader range of serovars has been included in microscopic agglutination test (MAT) serology for canine leptospirosis. It also may have resulted from increased contact between dogs and the reservoir hosts for these serovars.^2^ The pathogenic relevance of the serovar classification has been problematic, because both pathogenic and nonpathogenic leptospires can belong to the same serovar, presumably as a result of transfer of genes determining serotype among different species.^4^,^15^

The need for a Consensus Statement was suggested at the 2008 ACVIM Forum Infectious Disease Study Group (IDSG) meeting for several reasons: increasing recognition of canine leptospirosis associated with development of antibodies to previously unrecognized serovars, studies suggesting poor prediction of the infecting serovar by the MAT and interlaboratory variation in test results, concerns regarding the risk of zoonotic transmission, and questions regarding vaccine safety and efficacy. During the 2009 ACVIM Forum, the IDSG sponsored an interactive session led by Drs Sykes, Goldstein, Lunn, Moore, and Hartmann. The ACVIM Board of Regents then selected the topic for a Consensus Statement, and identified a chairperson (Sykes) and panel members. The panelists used the available literature to present evidence-based justification for recommendations on which there appeared to be a consensus. A draft was presented verbally at the 2010 ACVIM Forum, and a written draft was posted on the ACVIM web site for comments by the membership before submission of a revised manuscript to the ACVIM Board of Regents and the editors of the *Journal of Veterinary Internal Medicine*.

## What Clinical Syndromes Are Associated with Canine *Leptospira* Infection?

Infection of dogs with leptospires results in illness of varying severity, depending on the infecting strain, geographical location, and host immune response. Some dogs display mild or no signs of disease, whereas others develop severe illness or death, often as a result of renal injury. In general, veterinarians should suspect leptospirosis in dogs with signs of renal or hepatic failure, uveitis, pulmonary hemorrhage, acute febrile illness, or abortion.

Fever occurs early in the course of illness, and may be accompanied by shivering, generalized muscle tenderness, and reluctance to move. Dogs presenting with acute renal failure may show polyuria, polydipsia, dehydration, vomiting, diarrhea, inappetence, lethargy, or abdominal pain or some combination of these signs.^13^,^16^–^20^ Oliguria or anuria also may occur. Dogs may present with signs of hepatic failure, including icterus.^19^,^20^ Other reported manifestations of infection include conjunctivitis,^18^,^21^ uveitis,^22^^,^^a^ and tachypnea or dyspnea because of acute respiratory distress syndrome or leptospiral pulmonary hemorrhage syndrome (LPHS), which has been reported most frequently in dogs from some parts of Europe.^19^,^20^,^23^^,^^b^ LPHS is increasingly recognized in human patients, appears to have an immune-mediated basis, and is associated with high mortality.^4^,^24^,^25^ Pathologic lesions in dogs resemble those described in humans.^20^ Changes suggestive of pancreatitis have been detected in some dogs by abdominal ultrasonography. Hematuria can occur after natural and experimental infection.^19^,^20^ Bleeding tendencies also may be manifested as hematemesis, hematochezia, hemoptysis, melena, epistaxis, and petechial hemorrhages.^19^,^20^,^21^,^26^ The pathophysiologic mechanisms of bleeding in dogs and humans with leptospirosis are incompletely understood.^27^ Hepatic failure, disseminated intravascular coagulation (DIC), and direct vascular damage by spirochetes may play variable roles. Vasculitis also may be manifested as peripheral edema and mild pleural or peritoneal effusion. Meningitis is recognized in people with leptospirosis, but has not been documented in dogs. Cardiac damage occurs in human patients^28^ and ECG alterations suggesting myocardial damage can occur in dogs.^26^ Abortion has occurred in dogs after transplacental spread of serovar Buenos Aires,^29^ and 1 report suggested abortion associated with serovar Bratislava infection.^30^

Polyuria and polydipsia can develop in dogs with leptospirosis in the absence of azotemia. In some cases, this may result from a decreased glomerular filtration rate sufficient to cause impaired renal concentrating ability. However, these patients also may be hyposthenuric.^19^,^31^ Experimentally, leptospiral infection causes decreased vasopressin responsiveness of the inner medullary collecting ducts,^32^ suggesting polyuria may result from acquired nephrogenic diabetes insipidus.

Chronic active hepatitis was reported in 1 kennel in association with development of antibodies to serovar Grippotyphosa^33^ and in another to serovar Australis.^34^ Attempts to detect leptospiral DNA in liver samples from dogs with chronic hepatitis were unrewarding.^35^ Leptospirosis should be considered as a differential diagnosis in dogs with hepatitis or hepatic fibrosis. Similarly, dogs surviving acute renal tubulointerstitial injury may have residual chronic kidney injury that progresses over months to years, culminating in signs of decompensated chronic kidney disease.

Attempts have been made to correlate the infecting serovar with clinical presentation.^13^,^14^ Evidence for a correlation has been weak because of the poor ability of antibody tests to predict the infecting serovar, and lateral transfer of virulence attributes may occur between serovars.^4^,^15^ Thus at this time, no clear correlation has been made between the suspected infecting serovar based on antibody testing and clinical manifestations of disease in naturally occurring canine leptospirosis. No associations between clinical manifestations and infecting serovars have been detected in human patients,^1^ and multiple serovars have been isolated from humans in some outbreaks.^36^ Future attempts to correlate clinical presentation and infecting leptospiral strain should be based on a combination of isolation, serotyping, and genetic studies.

## Does Leptospirosis Occur in Cats?

Although serologic evidence of exposure of cats to leptospires exists,^37^–^39^ clinical disease in cats is rarely reported.^40^–^42^ Serovars Canicola, Grippotyphosa, and Pomona have been isolated from cats. Experimental infection of cats results in leptospiremia and leptospiruria, but disease is generally mild,^43^–^45^ although histopathologic evidence of renal and hepatic inflammation can be present. Cats may be exposed as a result of rodent contact.^37^ The extent to which cats contaminate the environment with leptospires is unknown.

## What Is the Geographic Distribution of Leptospirosis in Dogs?

Leptospirosis is especially prevalent in geographic regions with higher annual rainfall and warm climates, but factors such as host exposure and the presence of wild and domestic animal reservoir hosts also influence geographic distribution of the disease. For humans, the Caribbean and Latin America, the Indian subcontinent, Southeast Asia, Oceania, and to a lesser extent Eastern Europe are major disease foci. Hawaii accounts for most human cases in North America.^46^ Within the United States, regions of high antibody prevalence (titers ≥1,600) among dogs include Hawaii, the west coast (especially northern California, Oregon, and Washington), the upper Midwest and Midwest parts of Texas, Colorado, and the northeast and mid-Atlantic coastal regions (unpublished observations).^47^ Canine leptospirosis also occurs in the southeastern United States.^48^,^49^

## What Risk Factors Predispose Dogs to Leptospirosis?

Pathogenic leptospires infect and are shed from the renal tubules of a wide range of domestic and wild animals. Naïve animals become infected by contact of intact mucous membranes or abraded skin with infected urine or urine-contaminated soil, water, food, or bedding.^1^,^4^ Leptospires do not replicate outside of the host but may remain viable for weeks to months in soil saturated with urine.^50^ Transmission also has occurred after bite wound inoculation, ingestion of infected tissues, and venereal and placental transfer. In 1 study from Thailand, ingestion of raw meat by dogs was associated with leptospirosis.^51^

The organism prefers temperatures around 30°C, and freezing and UV radiation inactivate leptospires. Contact with slow-moving or stagnant warm water is a risk factor in humans.^52^–^55^ Outbreaks of disease in dogs have followed periods of higher rainfall,^16^,^56^ with overall peak seasonal distribution occurring in the late fall in the United States.^57^ The time of year for peak leptospirosis incidence varies geographically depending on local rainfall patterns and periods of freezing temperatures.^a^ In some geographic regions, disease generally occurs in dogs that are exposed to or drink from rivers, lakes, or streams,^58^,^59^ or dogs roaming on rural properties. In others, suburban backyard dogs may be exposed after contact with urbanized wild animal populations. In developing countries, access to sewage increases risk of the disease in dogs.^51^ Outdoor, intact male working dogs have been shown to be at risk,^60^ although dogs of any age, breed, and sex may become infected. In areas where wild animal species access suburban backyards, small breed dogs with minimal contact with water sources may be at risk. Contact with rodents also may pose a risk to these dogs, as well as dogs residing within cities.^61^

## What Is the Incubation Period for Leptospirosis in Dogs?

The incubation period for leptospirosis can be as short as a few days, the organisms replicating rapidly within the blood as early as 1 day after infection before invading tissues.^62^ The incubation period in experimental studies has been 7 days, but varies depending on the infecting dose, strain, and host immune response.^17^ Shorter incubation periods can occur with large inocula, and longer incubation periods may occur after low-grade, chronic infections of the renal tubules or hepatocytes,^63^,^64^ with clinical illness not being detected until some time after renal or hepatic injury.

## What Serovars Cause Disease in Dogs?

Infecting serovars vary geographically among dog populations depending on exposure to infected wild or domestic animal reservoir hosts. A complete understanding of infecting serovars in the dog population has been limited because published studies usually have not included isolation efforts. Furthermore, the MAT has poor ability to predict the infecting serogroup.

Although dogs are considered maintenance hosts for leptospires in serogroup Canicola, the prevalence of seroreactivity to this serogroup in dogs from the United States and Europe currently is low. Infection with serogroup Canicola serovars was reported in dogs from the United States in the 1950s and 1960s.^65^,^66^ Chronic canine infection with leptospires may be an ongoing public health problem in developing countries.^67^–^69^ Serovar Icterohaemorrhagiae, the major serovar infecting humans worldwide,^4^ is often found in rodent populations and was isolated from dogs in the United States before 1980.^70^–^72^ Based on antibody testing, evidence of canine infection by this serovar is less common now, possibly because of improved rodent control and other public health measures in cities and on farms in the United States. A high prevalence of seroreactivity to serogroup Icterohaemorrhagiae sometimes has been documented in nonrodent periurban wildlife (eg, raccoons^73^), and the role of transmission of serovar Icterohaemorrhagiae by these hosts is unclear. Given that the MAT is not effective for predicting the infecting serovar, more studies by isolation are required to confirm the importance of various wildlife hosts as reservoirs of serogroup Icterohaemorrhagiae.

Serovars from serogroups Icterohaemorrhagiae and Canicola cause disease in dogs after experimental inoculation.^21^,^74^–^79^ A study from India reported disease in dogs experimentally infected with serovars Autumnalis and Canicola.^62^ An Autumnalis serovar was isolated from sick dogs in France.^80^ A Pomona serovar caused disease after experimental inoculation of dogs.^17^ In the same study, inoculation of dogs with a serovar Bratislava isolate did not result in disease or seroconversion. However, another Bratislava serovar was isolated from a dog with leptospirosis.^81^ Antibody titers to serovar Bratislava (serogroup Australis) often increase with titers to Grippotyphosa and Pomona, and thus in some cases serologic evidence of serovar Bratislava exposure may represent cross-reactivity. Serovar Grippotyphosa also causes disease in dogs,^18^,^48^ and other studies have reported disease in association with serogroups Sejroe^82^ and Ballum.^49^ Although an Autumnalis serovar was isolated from raccoons in Georgia in the 1950s, the panel is unaware of any serovar Autumnalis isolates from dogs in North America. Antibody titers to serovar Autumnalis often increase together with antibody titers to serovars Grippotyphosa, Pomona, and Bratislava. Nonspecific increases in titers to serogroup Autumnalis have been observed in dogs with diseases other than leptospirosis, and in dogs vaccinated for or infected with serogroup Pomona or Grippotyphosa (unpublished observations), and caution is advised when interpreting titers to this serovar.

## What Clinicopathologic Abnormalities Are Expected in Dogs with Leptospirosis?

Renal tubular infection by leptospires is associated with acute interstitial nephritis and tubular dysfunction, although acute tubular necrosis can occur in naturally infected dogs.^20^ Mesangial proliferative glomerulonephritis and interstitial nephritis were associated with seropositivity in 1 study, but the dogs evaluated were strays and confounding factors, such as coinfections may have existed.^9^ Histopathologic changes in the liver often are mild and can include mild to moderate scattered hepatic necrosis^20^ and mild neutrophilic periportal hepatitis.

Findings on CBC may include neutrophilia, sometimes with a left shift, lymphopenia, and mild to moderate, nonregenerative anemia. Uncommonly, severe anemia occurs,^21^ which may follow gastrointestinal or pulmonary hemorrhage. In contrast to what is observed in cattle, hemolysis does not appear to be a feature of canine leptospirosis. Thrombocytopenia is present in up to 58% of affected dogs,^13^,^14^,^19^,^20^,^26^ and when accompanied by evidence of acute kidney damage with or without hepatic injury, can help increase suspicion for a diagnosis of leptospirosis.

Increased serum urea and creatinine concentrations may be present in >80–90% of dogs,^13^,^19^,^20^,^26^^,^^a^ although in 1 European study, increased serum creatinine concentration was present in only 57% of affected dogs.^14^ Hepatic dysfunction may be manifested by increases in serum ALT, AST, and ALP activities and total bilirubin concentration, almost always in conjunction with azotemia.^13^,^14^,^19^,^20^,^26^^,^^a^ Increases in serum ALP activity and total bilirubin concentration are more common than increases in the activity of serum ALT.^2^,^13^,^19^,^20^,^31^,^48^,^83^ A combination of azotemia and increased liver enzyme activities should markedly increase suspicion for leptospirosis. Electrolyte abnormalities may be a consequence of gastrointestinal or renal fluid losses. Inhibition of Na^+^-K^+^ ATPase activity within the nephron by leptospiral endotoxin may contribute to renal losses.^84^ Hyponatremia, hypochloridemia, marked hypokalemia, and hyperphosphatemia occur in many cases, but dogs with oliguric or anuric renal failure may become hyperkalemic. Leptospires are known to induce hypokalemic, nonoliguric renal failure in humans as a consequence of impaired tubular sodium reabsorption,^85^ and the same may occur in dogs.

Increased serum creatine kinase activity also may be present, presumably because of myositis.^26^ Increased serum troponin concentrations in some dogs suggest myocardial damage.^26^

Urinalysis from dogs with leptospirosis may show isosthenuria, occasionally hyposthenuria. Glucosuria and proteinuria are common in some geographic locations,^20^ and bilirubinuria, hematuria, pyuria, and cylindruria also may be present.^13^,^20^,^26^ Although high-molecular weight proteins have been detected in urine from some dogs with leptospirosis,^26^ results of another study suggested proteins were primarily tubular, rather than glomerular, in origin.^86^ Leptospires are not visible in the urine sediment by routine light microscopic evaluation.

Clotting function assays in dogs with leptospirosis show variable increases in fibrinogen, D-Dimer and fibrinogen degradation product concentrations, and decreases in antithrombin activity, in addition to thrombocytopenia.^26^,^87^ Prolongations of PT or PTT have been detected 6–50% of tested dogs.^13^,^20^,^26^ Prolongations were most prevalent in western European dogs.^20^ A shortened PT also may be present, possibly because of DIC.

Thoracic radiographs from dogs with leptospirosis may show diffuse interstitial patterns or more severe nodular interstitial to alveolar patterns.^20^,^23^^,^^b^ Abdominal sonography can disclose nonspecific findings including renomegaly, increased cortical echogenicity, perirenal fluid accumulation, mild pyelectasia, and a medullary band of increased echogenicity.^88^ Enlargement and hypoechogenicity of the pancreas, thickening of the gastric and (less commonly) intestinal wall, splenomegaly with a mottled splenic echotexture, and mild abdominal lymphadenomegaly also may be found.

## How Should Antibody Testing Be Used to Diagnose Canine Leptospirosis?

Use of antibody testing for diagnosis of leptospirosis generally is based on the MAT, which involves reacting serial dilutions of patient sera with an array of live leptospiral serovars, and assessment of organism agglutination by darkfield microscopy. The highest serum dilution causing agglutination of 50% of the leptospires in the reaction is reported to the veterinarian. The MAT is widely available and inexpensive, and there is a large body of data regarding its use; as such, it is the current diagnostic test of choice for canine leptospirosis in patients with consistent clinical signs. Unfortunately, the test is hazardous to perform because of the need to maintain live cultures of pathogenic serovars, and is difficult to standardize. Test interpretation is somewhat subjective and requires considerable expertise, and serovar identity must be verified regularly to ensure accurate results.^89^,^90^ Serovar cultures may become cross-contaminated over time. Considerable variation in results has been noted among laboratories performing the MAT for diagnosis of canine leptospirosis, possibly as a result of variable quality control and standardization.^c^ There is a lack of consensus over what titer should be used as a cut-off for a negative result. The International Leptospirosis Society offers an inexpensive leptospirosis testing proficiency scheme that enables laboratories to maintain quality assurance for the MAT on a regular basis.^89^,^91^ Laboratory participation in the scheme is encouraged and it is recommended that practitioners use laboratories that participate in this program. Molecular methods also have been advocated to ensure quality control within leptospiral reference laboratories.^92^

In the 1st week of illness, dogs frequently have negative MAT results, and consequently acute and convalescent phase antibody testing is recommended. Traditionally, convalescent titers for acute infectious disease diagnosis are performed 2–4 weeks after the acute titer, although seroconversion can occur as early as 3–5 days after dogs are brought to a veterinarian. Practitioners should wait 7–14 days between successive titers to demonstrate seroconversion. A 4-fold change in titer supports recent infection, although an increase in titer may be blunted by antimicrobial therapy. Titers resulting from previous vaccination, exposure, or chronic infection generally change more slowly or not at all. Titers can persist for at least 1 year after natural infection, and in 1 study, generally declined by 4 months after vaccination.^93^ Postvaccinal titers may persist for longer and be maintained at high levels if ongoing exposure to field strains occurs. Thus, although single positive titers can increase suspicion for the disease, even when high (≥800), they do not confirm a diagnosis of leptospirosis. This is especially important in dogs with a history of vaccination, because although postvaccinal titers tend to be low, high titers (≥1,600) have the potential to persist after vaccination, and cross-reactivity to nonvaccinal serogroups can occur.^93^ In 1 study, the sensitivity of a single MAT titer ≥800 for diagnosis was 22–67%, depending on the laboratory used, and the specificity was 69–100%.^d^

False negative titers may occur if the infecting serovar is not including in the panel of serovars used to perform the test. MAT tests used for diagnosis of human leptospirosis generally include a larger panel of serovars (>20) than those used for veterinary diagnostic testing (5–7 serovars). MAT assays for canine leptospirosis should include serovars known to be circulating in the local dog population, although this information is not always readily available.

## What Are the Limitations of Antibody Testing for Understanding the Epidemiology of Canine Leptospirosis?

The MAT is a serogroup- rather than a serovar-specific test, because antibodies to serovars within the same serogroup cross-react extensively.^1^ Because of shared antigens, some cross-reactivity among different serogroups also occurs after human and canine exposure to leptospires. In the past, the serogroup with the highest titer has been interpreted as the infecting serogroup. However, studies of infected humans with culture-proven infections have shown that accurate prediction of the infecting serovar occurs in <50% of cases.^94^ Higher, cross-reactive titers can occur to a noninfecting serovar. These so-called “paradoxical reactions” are especially common in early infection, and when multiple serovars circulate within the population.^1^,^69^ Furthermore, the MAT used to perform studies in humans included approximately 20 serovars, and the serovars included in the test reflected those circulating in the population.^94^ Even lower specificity could be predicted when the number of serovars included in the test is small and not reflective of actual serovars infecting the dog population in a specific geographic region.

The predicted identity of the infecting serogroup also has been shown to change over the course of infection in dogs.^c^ Previous vaccination can influence the pattern of serovar reactivity.^93^ In dogs, the serogroup with the highest titer also varies depending on which laboratory performs the MAT, again likely reflecting the lack of standardization of the assay.^c^ Thus, the results of the MAT are not recommended to predict serogroups circulating in the dog population. Instead, studies involving isolation of leptospires from dogs are recommended for epidemiological purposes, as well as for selection of antigens for diagnostic assay development and vaccine design.

## How Should Polymerase Chain Reaction (PCR) and Culture Be Used to Diagnose Canine Leptospirosis?

Culture and PCR detect pathogenic leptospires or their nucleic acid, respectively, and have potential utility early in the course of untreated infection when antibody assays are frequently negative and antimicrobials have not yet been administered. They also can confirm active infection in animals with positive antibody test results that have a history of vaccination with leptospiral vaccines, because previous vaccination should not yield false positive results by these methods.^e^ They may detect infection in dogs with chronic renal or hepatic disease.

In the first 10 days of infection, organism numbers are highest in blood, and thus blood is the sample of choice during the first week of illness.^17^ After that time, organisms are present in highest concentration in urine. When the time of infection is unknown, simultaneous testing of blood and urine may increase diagnostic sensitivity. Recent antimicrobial treatment can result in false negative test results for both culture and PCR, although multiple doses of antimicrobials may be required before PCR becomes negative, because PCR detects both viable and nonviable organisms.

Culture of leptospires requires special media.^f^ Organisms may be destroyed during transport to the laboratory. The growth of leptospires is slow, requiring incubation for up to 3–6 months, and consequently culture is not useful for early diagnosis. Overgrowth with other bacteria may occur in contaminated cultures. For optimal sensitivity, venous blood should be collected by aseptic technique and immediately inoculated (alongside the patient) into blood culture bottles containing culture medium for *Leptospira* before sending the inoculated bottles to the laboratory.^95^ After the 1st week of illness, urine should be collected by cystocentesis, and 1 drop of urine should be inoculated into 5 mL of culture medium within 2 hours of collection. Cultures must be performed by laboratories with expertise in isolation and identification of leptospires, and the commercial availability of leptospiral culture is not widespread. However, a proper understanding of the epidemiology of leptospirosis depends upon serotyping and genetic typing after isolation, and thus attempts to isolate leptospires from dogs with leptospirosis are encouraged.

PCR assays for detection of leptospiral nucleic acid are increasingly offered by commercial veterinary diagnostic laboratories worldwide. Both conventional and real-time assays have been developed, and their use has been reported.^20^,^96^–^99^ Although PCR assays have been designed to detect only pathogenic leptospiral serovars, currently available assays do not differentiate between serovars or serogroups and thus are not useful for studying the epidemiology of leptospiral strains. Recent reports suggest that PCR typing methods may be used to some extent to identify infecting serovars.^100^ Not all PCR assays are alike and they vary considerably in their performance. Negative results do not rule out leptospirosis, because they may occur when organism numbers in a sample are low, or other factors, such as PCR inhibitors, are present. Currently, there is limited information regarding the validity of PCR assays for detection of pathogenic leptospires infecting dogs, as well as their sensitivity, specificity, and positive predictive value, and so positive and negative test results should always be interpreted in conjunction with other diagnostic methods such as acute and convalescent phase antibody testing. The sensitivity of 1 assay on blood was greatest when whole blood, as opposed to sera, was used. Sensitivity was 90% in the first 5 days of illness, after which it decreased to 50% (Riediger et al, manuscript in preparation). Because apparently healthy dogs may shed leptospires, a positive PCR test result on urine may not necessarily correlate with illness but is useful to identify a chronic carrier state.^99^,^101^

Other methods that can be used to confirm the presence of *Leptospira* spp. in tissues include silver staining of biopsy or necropsy specimens (which lacks sensitivity), immunohistochemistry, tissue PCR, and in situ hybridization.^102^

## Are Other Diagnostic Assays for Canine Leptospirosis Needed?

Given the insensitivity of the MAT in the 1st week of illness, the hazardous and subjective nature of the MAT, the potentially fatal consequences of inadequate therapy, and the zoonotic risks relating to handling dogs with leptospirosis, assays having high sensitivity early in the course of illness and a rapid turn-around time have clear benefit. Although nucleic acid-based assays have the potential to fulfill this need, low-cost rapid assays for IgM or leptospiral antigen that could be performed as point-of-care tests would be useful. An IgM immunoblot assay was 88% sensitive in the first 3 days of human leptospirosis compared with 2% for the MAT.^103^ Use of rapid, broadly reactive antibody assays as screening tests before performing the more specific and cumbersome MAT may help decrease false negative test results relating to inadequate serovar inclusion in the MAT and negate the need for subsequent MAT testing in dogs that test negative by screening assays. New antibody assays should be validated in the geographic region in which they are to be used, because sensitivity may be affected by regional differences in serovar prevalence. Ideally, they should detect antibodies that react only with pathogenic serovars. Recombinant LipL32-based assays recently were evaluated and found to be sensitive and specific in dogs and humans compared with MAT testing.^104^,^105^

Rapid and sensitive assays for organism detection that allow subsequent organism typing by molecular methods also are needed for epidemiologic studies and vaccine design. Ideally, these assays should allow prediction of the serovar present, reservoir preferences, and virulence attributes.

## What Antibiotics Should Be Used for Treatment of Canine Leptospirosis?

The role of antimicrobial therapy in the treatment of human leptospirosis has been controversial.^106^,^107^ Treatment initiated after 4–7 days of illness is less effective in promoting clinical recovery. Nevertheless, the World Health Organization recommends that all human patients diagnosed with leptospirosis be treated with antimicrobial drugs.^95^ Treatment for 7 days has been used.^108^–^110^

The optimal treatment for leptospirosis is unknown. Penicillins or doxycycline traditionally have been the antimicrobials of choice for treatment of humans and dogs with leptospirosis.^107^,^108^ Ceftriaxone and cefotaxime are as efficacious as penicillin in human leptospirosis.^107^,^109^ Azithromycin also may be effective.^110^ First generation cephalosporins appear less effective, and leptospires are resistant to chloramphenicol.^111^ The use of fluoroquinolones has been controversial. Efficacy similar to doxycycline in hamsters has required doses ≥25 mg/kg/d.^112^ In 1 study that used quantitative PCR for leptospiral detection, ofloxacin was unable to clear leptospires from the kidneys and blood of a hamster model, ampicillin did not clear organisms from the kidney, but doxycycline cleared organisms from all sites within 3 days of infection.^113^ Orbifloxacin was ineffective in 1 dog with leptospirosis that responded to amoxicillin.^114^

Based on these data, the consensus panel recommends treatment of canine leptospirosis with doxycycline, 5 mg/kg PO or IV q12h for 2 weeks, but the optimal duration of antimicrobial therapy requires further investigation. Treatment should not be delayed pending results of diagnostic testing for leptospirosis. If vomiting or other adverse reactions preclude doxycycline administration, dogs with leptospirosis should be treated with ampicillin, 20 mg/kg IV q6h, with dose reduction for azotemic dogs. Penicillin G (25,000–40,000 U/kg IV q12h) also could be used. Ampicillin should not be administered orally because it is not reliably absorbed from the gastrointestinal tract.^115^ Dogs should receive doxycycline for 2 weeks after gastrointestinal signs abate in order to eliminate organisms from the renal tubules. Concurrent fluoroquinolone use is not recommended in dogs with leptospirosis because it contributes to antimicrobial resistance in other bacteria. Additional prospective studies that evaluate clearance of organisms from the blood and urine with different antimicrobials are indicated in dogs.

## When Should Dialysis Be Recommended for Treatment of Canine Leptospirosis?

Renal replacement therapy with intermittent hemodialysis or continuous renal replacement therapy (CRRT) can be life-saving for many dogs with severe anuric leptospirosis.^16^^,^^a^ More than 80% of patients that would otherwise die from the consequences of severe uremia survive with supportive renal replacement therapy. Recovery of adequate renal function usually occurs within 2–4 weeks of starting dialysis. Sometimes only 1–3 treatments are required before polyuria ensues and renal function begins to recover. Early hemodialysis has been associated with increased survival and shorter hospital stays in human patients with leptospirosis.^116^ Renal replacement therapy is indicated in dogs with inadequate urine output that are developing volume overload, hyperkalemia, BUN > 80 mg/dL, or signs of uremia that are not responsive to medical management. Increased availability of hemodialysis will help more dogs survive this disease. Practitioners should consider early referral of dogs failing to produce adequate urine volume despite proper fluid therapy to centers with intermittent hemodialysis or CRRT facilities when client finances allow.

## How Should LPHS in Dogs Be Treated?

Dogs with LPHS may require oxygen therapy and, if severe, mechanical ventilation. Studies of human patients with LPHS showed improved outcome after cyclophosphamide therapy and plasma exchange.^25^,^117^ Whether dogs with LPHS would also benefit from cyclophosphamide treatment remains to be determined. Treatment of humans and dogs with respiratory complications with dexamethasone and desmopressin has not improved outcome.^118^^,^^g^

## What is the Expected Response to Therapy?

Provided severe respiratory complications are absent, the prognosis for dogs treated early and aggressively in the course of leptospirosis with appropriate antimicrobial drugs and IV fluids, with or without diuretics, is good, especially when intermittent hemodialysis is available. Survival rates of approximately 80% have been reported, both among dogs treated conservatively and those treated with dialysis,^13^,^16^ although virtually all dogs that are dialysis dependent and do not receive dialysis would be expected to die. The prognosis for dogs developing severe respiratory complications is poorer.^20^^,^^b^^,^^f^ A high prevalence of respiratory complications contributed to overall mortality rates of 48 and 36% in 2 studies from western Europe, respectively.^20^^,^^b^

Successful treatment is associated with gradual return of serum urea and creatinine concentrations to reference ranges within 10–14 days, although regeneration of damaged renal tissue may continue for over 4 weeks after treatment of infection. The bilirubin concentration may decline more slowly than the activities of serum ALT and ALP. Platelet counts often improve within 1 week of initiating antimicrobial treatment. In some dogs, especially those treated late in the course of illness, permanent residual kidney damage may occur. IV fluid therapy should be tapered gradually before being discontinued, to ensure that polyuria is resolving and the patient is able to drink sufficient water to maintain hydration. Prolonged inappetence may require nutritional support by enteral or parenteral routes.

## What Clinicopathologic Variables Should Be Monitored during Treatment for Canine Leptospirosis?

Dogs with acute leptospirosis ideally should have serum biochemistry panels performed every 24 hours during hospitalization to monitor renal function, liver enzyme activities, serum protein concentrations, and electrolyte and acid-base status. More frequent monitoring may be indicated for dogs with marked electrolyte and acid-base derangements. PCV should be monitored every 24 hours, and the CBC every 48 hours during hospitalization.

Dogs with nonoliguric renal failure may be profoundly polyuric. Fluid therapy may be provided by the “outs and ins” method. Some patients may require fluid rates >200 mL/kg/d. For dogs with oliguric or anuric renal failure, close attention should be paid to urine output by use of a closed, indwelling urinary catheter and collection bag system. Initially, urine output should be monitored at least hourly. Referral to a 24-hour care facility is recommended if adequate time for monitoring is not available in the practice. Indwelling, rather than intermittent, urinary catheterization is recommended for dogs requiring urinary catheterization because of decreased risk of exposure to infected urine with indwelling catheterization. Serial physical examinations with frequent monitoring of body weight, respiratory rate, lung sounds, blood pressure, and, if possible, central venous pressure are indicated to assess for early signs of overhydration that might necessitate diuretic therapy or dialysis. Once a patient is appropriately hydrated, fluid administration must be adjusted to prevent life-threatening fluid overload.

Although follow-up will vary from dog to dog, at a minimum, dogs should be reexamined no later than 1 week after discharge from the hospital, and a serum biochemistry panel and urine specific gravity should be performed. If thrombocytopenia or anemia were present during hospitalization, a CBC could also be performed. This also represents an opportunity to obtain convalescent antibody titers.

## What Are the Public Health Implications of Canine Leptospirosis?

Leptospirosis in humans occurs after an incubation period of 2–25 days, and varies in severity.^1^,^4^,^95^ Infection in some humans is subclinical. Others develop a mild, influenza-like illness. The most severe manifestations of leptospirosis in humans are hepatic and renal failure (Weil's disease) or LPHS. Weil's disease typically occurs 1 week after recovery from an initial febrile illness that is accompanied by myalgia, headache, chills, and conjunctivitis.^1^

The public health implications of canine leptospirosis vary geographically. In developing countries, stray dogs may represent a reservoir of infection for humans,^67^ although rodents also may play a role.^69^,^119^ In developed countries, most leptospirosis cases in humans result from recreational activities involving water.^53^,^55^,^120^ Individuals that contact farm animals are also at risk.^52^,^121^ In 1 study, 10% of 61 leptospirosis cases in humans in California over the last 20 years resulted from pet contact.^122^ Contact with adopted wild rodents also has resulted in human disease.^123^,^124^

In general, animals developing acute leptospirosis are incidental hosts and do not develop a chronic carrier state. Transmission from incidental hosts to other animals is rarely reported,^4^ and the few reports^72^,^125^–^127^ suggesting transmission of leptospirosis from pet dogs to humans have not been substantiated by molecular methods. The extent of shedding of specific leptospiral serovars by dogs after infection requires evaluation. Anecdotal evidence suggests it is difficult to detect leptospires in the urine of dogs receiving penicillin or doxycycline treatment, and thus appropriate antimicrobial therapy may also decrease the possibility of zoonotic transmission. Nevertheless, the full impact of antimicrobial therapy on leptospiral shedding from infected dogs requires further study. Positive PCR results detected in animals receiving antimicrobial therapy may reflect nonviable organisms, which would not be a zoonotic risk.

## What Recommendations Should Be Made to Minimize the Risk of Zoonotic Transmission in the Hospital?

Having a high index of suspicion for leptospirosis in dogs with renal injury and handling them appropriately may decrease zoonotic transmission of leptospires in the hospital. All dogs with acute renal failure, including “acute-on-chronic” renal failure, should be managed as leptospirosis suspects until an alternate diagnosis has been made. Based on rodent model studies, viable organisms are most likely to be present in blood or urine before initiating antimicrobial therapy, and within the first 2–3 days of treatment.^113^ Leptospires might be shed for months in urine if appropriate antimicrobial treatment is not initiated. Leptospires generally survive poorly in the environment and are susceptible to UV irradiation, dessication, and routine disinfectants, although the degree to which organisms could survive in urine-soaked hair is unknown. Caution is recommended when handling dogs suspected to have leptospirosis. The movement of dogs suspected to have leptospirosis around the hospital should be minimized, and areas of contact should be disinfected. Warning labels should be placed on cages of dogs suspected to have leptospirosis, and pregnant or immunocompromised humans should avoid contact with these patients. Because many of these dogs are critically ill and require frequent monitoring, and leptospires are not readily transmitted between dogs, housing in isolation is not necessary. If possible, patients should be placed in floor-level cages and housed away from high traffic areas. Care should be taken to avoid needle-stick injuries and other blood contact. Gloves and a disposable gown should be worn, and either protective eyewear and a facemask, or, alternatively, a full face shield should be worn if aerosolization of urine is possible, such as when manipulating urinary catheters or collection systems, or when cleaning areas of urine spillage.^128^ Pressure washing of runs should be avoided as it may contribute to urine aerosolization. An indwelling urinary catheter should be placed if urine output requires monitoring, or if urinary incontinence is present, to minimize urinary contamination of the environment. If a urinary catheter is not in place, dogs should be walked frequently enough that urination does not occur in the hospital, and preferably by a route that avoids common hallways. Patients also could be moved through the hospital on a gurney. They should be allowed to urinate in a restricted area, preferably one that can be easily and immediately decontaminated, such as on a hard, nonpermeable surface that is free of organic matter. If urine spills occur, they should be disinfected and cleaned immediately. Bathing of hair that becomes soaked in urine is recommended. Hand washing should be performed before and after handling each patient after glove removal, and cages should be thoroughly cleaned and disinfected daily. Gloves, a disposable gown, and face protection should be worn when handling soiled bedding and when cleaning cages or runs. Normal laundering of soiled bedding will inactivate leptospires, but individuals handling the bedding should wear protective clothing. Disposable bedding should be placed in biohazard bags and handled appropriately.

Urine collected from dogs with leptospirosis can be inactivated with disinfectant solutions (eg, 1 : 1 aqueous dilution of 10% bleach solution) and should be disposed of properly. Iodine-based disinfectants, accelerated hydrogen peroxide, and quaternary ammonium solutions also are effective. In dogs with indwelling urinary catheters, disinfectant should be injected directly into the collection bag before disposing of the urine. In designated outdoor areas where infected dogs have urinated, treatment of the area with 10% bleach solution is recommended. All blood, urine, and tissues from dogs suspected to have leptospirosis should be treated as medical waste, and the handling of such material may vary depending on local regulations.^129^ If a dog dies or is euthanized, individuals handling the remains should be alerted of the zoonotic potential of the carcass.

All personnel that may have had direct or indirect contact with a dog suspected to have leptospirosis should be informed of the risks. These people include radiology personnel and laboratory personnel handling blood, urine, or tissue samples from patients.

Veterinarians should contact their local or state health department or the Centers for Disease Control and Prevention for guidance if additional questions arise regarding the public health risks and zoonotic transmission of leptospirosis.

## Should Dogs Living with Dogs Diagnosed with Leptospirosis Be Treated?

Subclinical seroconversion has been documented in some dogs living in the same household with dogs with leptospirosis, possibly as a result of common exposure. Because of the zoonotic potential of leptospirosis, treatment of other dogs in the household that may have been coincidentally exposed to a source of leptospires in the environment is recommended, ideally with monitoring of acute and convalescent phase antibody titers. The recommended treatment is doxycycline, 5 mg/kg PO q12h for 14 days.

## What Recommendations Should Be Made to Minimize the Risk of Zoonotic Transmission within the Home?

Treated dogs represent a low risk to household members. In addition, urinary shedding usually does not commence until 7–10 days after infection, and consequently dogs in the first few days of illness also may not represent a clinically relevant source of infection. Nevertheless, until proper antimicrobial therapy is completed, owners should avoid contact with their dog's urine and wear gloves when cleaning up urine. Routine household disinfectants should be used to clean areas of urine contamination in the home. Dogs should be taken to urinate and should urinate away from standing water, where no other animals and people, especially children, will have access. Owners should be advised to wash their hands after handling their pets. Upon diagnosis of leptospirosis, veterinarians should educate owners of the zoonotic potential of leptospirosis, and recommend they seek medical attention if illness occurs around the time their dog is diagnosed with leptospirosis or if they have questions about the disease in humans. Internet resources^128^ also can be provided. Immunocompromised humans should be referred to their medical practitioner for advice. Routine vaccination of dogs at risk of developing leptospirosis may decrease the risk of zoonotic transmission of the disease. Owners should be informed that their dog likely contracted leptospirosis by direct or indirect contact with wild or farm animals, which may represent ongoing risk factors.

## What Vaccines Currently Are Available for Canine Leptospirosis?

Currently, vaccines containing serovars Icterohaemorrhagiae, Canicola, Grippotyphosa, and Pomona are available in North America for prevention of canine leptospirosis. At the time of writing, bivalent vaccines containing Icterohaemorrhagiae and Canicola are available in other geographic locations including North America, Europe, South Africa, and Australia.

## How Effective Are Vaccines Against Canine Leptospirosis? Do They Prevent Development of the Carrier State? What Is the Duration of Immunity after Vaccination?

Current vaccines appear to effectively prevent disease resulting from experimental challenge and to a large degree prevent shedding caused by the serovars in the vaccine. They also protect for at least 12 months.^21^,^78^^,^^h^ Currently available bacterins elicit serogroup-specific immunity, but partial immunity to heterologous serogroups has been documented in some studies.^80^,^130^–^132^ Naturally occurring canine leptospirosis has been reported after vaccination with bivalent serovar Icterohaemorrhagiae and Canicola vaccines.^20^ The panel is unaware of leptospirosis in dogs that have been fully vaccinated with 4-serovar vaccines, but published data are lacking regarding the incidence of naturally occurring leptospirosis in such dogs. This may partly relate to the difficulty in definitively diagnosing leptospirosis in fully vaccinated dogs.

## What Adverse Effects Might Be Associated with Vaccination for Canine Leptospirosis?

Concern has been raised regarding the development of anaphylactoid reactions in dogs after leptospirosis vaccination, especially small breed dogs, although such reactions may occur in any breed. There is anecdotal evidence from veterinarians and industry that the prevalence of these reactions is decreasing, and may be similar to the rate induced by vaccines for other pathogens. In a study of acute vaccine reactions in dogs utilizing a large database, vaccines containing leptospiral antigen were no more reactive than other vaccines for dogs.^133^

## When Should Vaccination Be Recommended for Prevention of Canine Leptospirosis?

Annual vaccination with 4-serovar vaccines is recommended for at-risk dogs, regardless of breed, with the understanding that the definition of “at-risk” may vary geographically. In geographic locations in which infection occurs in urban, backyard dogs, all dogs may be at risk, and the vaccine may be considered part of a core vaccination protocol. In other locations, only dogs that contact wildlife, swim, hunt, or roam on farmland may be at risk.

## What Other Preventative Measures Can Be Recommended to Prevent Canine Leptospirosis?

Other methods of prevention include decreased access to potential sources of infection, such as marshy areas and standing water, and minimizing wild animal contact by use of fencing and rodent control.

## Can Dogs That Have Recovered from Leptospirosis Be Reinfected?

Evidence of recurrent leptospirosis in dogs after proper treatment is lacking. Nevertheless, annual vaccination for dogs that have recovered from leptospirosis could be considered, because such dogs are at risk of ongoing exposure, and whether or not life-long immunity results from natural infection is unknown. The duration of immunity in dogs after natural infection is likely to be at least as long as that induced by vaccination, and thus initial vaccination after recovery should occur 1 year after recovery. Although natural infection may elicit only partial cross-protective immunity to heterologous serogroups, dogs are most likely to be reexposed to a similar serovar to that involved in the initial infection. There is little evidence supporting the need for immediate boostering with a multivalent vaccine after recovery from infection. More studies are required to establish the true duration of immunity and degree of cross-protection among specific serovars after natural infection in dogs.

## Footnotes

a Sykes JE, Bryan J, Amstrong PJ. Comparison of clinical findings associated with canine leptospirosis between two teaching hospitals. J Vet Intern Med 2007;21:624 (abstract)

b Schweighauser A, Francey T. Pulmonary haemorrhage as an emerging complication of acute kidney injury due to canine leptospirosis. J Vet Intern Med 2008;22:1473 (abstract)

c Miller MD, Annis KM, Lappin MR, et al. Variability in the microscopic agglutination test for the diagnosis of leptospirosis in dogs. J Vet Intern Med 2007;21:624 (abstract)

d Miller MD, Annis KM, Lappin MR, Gill M, Lunn KF. Sensitivity and specificity of the microscopic agglutination test for the diagnosis of leptospirosis in dogs. J Vet Intern Med 2008;22:787 (abstract)

e Midence JN, Chandler AC, Goldstein RE. Assessing the effect of recent *Leptospira* vaccination on whole blood quantitative PCR testing in dogs. J Vet Intern Med 2010;24:763 (abstract)

f EMJH, Difco: http://www.bd.com/ds/technicalCenter/inserts/Leptospira_Medium_Base_EMJH.pdf. Accessed June 21, 2010.

g Schweighauser A, Francey T. Treatment of pulmonary haemorrhage in canine leptospirosis with desmopressin and dexamethasone. J Vet Intern Med 2008;22:1474 (abstract)

h Chandler AM, Goldstein RE. Assessing renal colonization in dogs 15 months after receiving a multi-serovar bacterin based on vaccine, versus unvaccinated dogs, all experimentally infected with *Leptospira kirschneri* serovar Grippotyphosa. J Vet Intern Med 2010;24:764 (abstract)

## Abbreviations

CRRT: continuous renal replacement therapy
DIC: disseminated intravascular coagulation
IDSG: Infectious Disease Study Group
LPHS: leptospiral pulmonary hemorrhage syndrome
MAT: microscopic agglutination test
PCR: polymerase chain reaction
